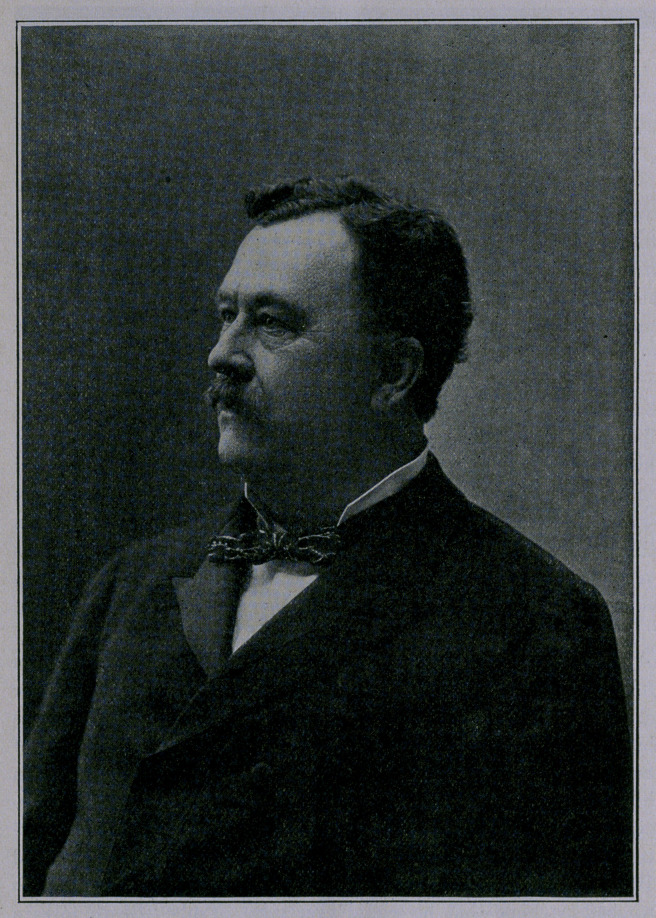# Dr. James W. McLaughlin

**Published:** 1909-12

**Authors:** 


### EDITORIAL DEPARTMENT.

### DR. JAMES W. McLAUGHLIN.

"THE BELOVED PHYSICIAN."

(Died November 13, 1909. Aged 69.)

The medical profession, not only of Texas but of the whole world, has lost one of its most learned, able and popular members in the death of this noted physician and scientist. We in Texas mourn today the loss of the foremost physician in the State. He stood out pre-eminently and conspicuous for his ability and his work. His indefatigable labors in the search for truth in the chemical and biological laboratories, his researches as to the causes of immunity and infection, and especially his discovery of the bacillus of dengue—all of which has been published and republished in the medical press of America and Europe, have given him a world-wide fame. Some of them have been incorporated in the text-books. His work will live, like that of many others who have caught glimpses of the truth and shed it abroad, even though the problems to which they set their hands remain as yet unsolved. To his dying day he retained not only his consciousness, but his thoughts dwelt on the subject of his last paper, "A Critical Analysis and Discussion of Ehrlich's Side-Chain Theory of Immunity." Although confined to his bed, where he had been for about three months, he discussed this paper with the writer clearly and intelligently, just seven days before he died. It was his Presidential Address, as President of the Texas Academy of Science. He had prepared it for his inaugural address, and in his enforced absence it was read by Dr. H. L. Hilgartner, and discussed by a number of members present, all of whom paid glowing tribute to the work and talent of their beloved Fellow and President-elect, lying at that moment upon a couch from which we had no hope that he would ever rise. Truly "Death loves a shining mark." Its arrows never reached the heart of a brighter, dearer or more beloved man. It had marked him for its victim a year or more ago, when the Mayos extirpated the entire cervical and maxillary glandular system in the desperate hope of arresting the dread cancer, which, beginning on the lip, spread downward. His paper—his favorite theme—"Theory of Immunity by Wave Interference and Catalysis"—as opposed to that of Ehrlich—had only recently appeared in the *New York Medical Record.* In recognition of his pre-eminent distinction and ability, Governor Campbell, in 1907, appointed him on the Board of University Regents. The flags were at half-mast on the Capitol and the University the day he was buried, Sunday, November 14th, while practically the entire citizenship of Austin crowded the church (St. David's, Episcopal) and filled the streets for several blocks. The grief and mourning of the family were shared by all, high and low alike, all of whom felt they had lost a personal friend and benefactor. Dr. McLaughlin practiced medicine in Austin thirty-two years, and until he was called to the Chair of Practice in Texas' great medical school, the Medical Department of the University, at Galveston. This chair he filled with distinction eight years, when he resigned and returned to Austin.

Dr. McLaughlin was President of the Texas State Medical Association in 1894. As a citizen he was beloved by all; as a man, almost idolized. They said that his presence in the sick room brought hope and healing; and as a friend he was simply delightful; loyal and true. The writer for more than a quarter century enjoyed his confidence, friendship and esteem, and its memory will ever be one of the most delightful of this fitful life's experiences. Genial, learned, witty, with a keen sense of humor, his face was always bright and smiling; joyous without levity, grave on occasion without solemnity. He was an optimist; a Christian gentleman. He died in the full hope and belief of a life beyond death's portals, in that

> "* * * * * * * sphere
> Where all is made right that so puzzles us here,—
> Where the glare and the glitter and tinsel of time
> Fade and die in the light of that region sublime;
> Where the soul disenchanted of flesh and of sense,
> Unscreened by its trappings and shows and pretense,
> Must be clothed for the life and the service above
> With purity, truth, faith, meekness and love—"

and he was prepared for it by a life of singular purity, goodness and usefulness to his fellow man. His death came in mercy, robbed of all its terrors; a beautiful, peaceful, painless and most enviable death. He died in a tranquil sleep. Perhaps it is better to be thus taken in the zenith of one's fame, in mid-career of usefulness, than to linger until old age shall have thrown its pall on him and decrepitude should have made him only a memory of what he had been. He died at 4:30 a. m., Saturday morning. Prior to composing himself for sleep, he had spent the early hours of the evening in pleasant, cheerful converse with his family, partaking of some candy sent in by a friend, and there was no mention made of or allusion to the end being so near or of the fact that soon these delightful relations would cease. Good night was said and he fell asleep, to awake in a celestial world. It is most regrettable that the writer of this poor tribute was absent from Austin and knew nothing of the death of his friend until it was too late to reach Austin in time to be present at the funeral.

Rev. Dr. Lee, rector of St. David's, a friend of nearly a half century, after reading the burial service, said:

" 'Consider the perfect man and behold the upright, for the end of that man is peace.'

"Beloved, these words come into one's mind when thinking of the life of this, our friend. I must leave it to his brother physicians to speak of his eminence in his chosen profession, and there is none calling for greater heroism and sacrifice and unwearied devotion. In this spirit he has spent his life. No man has stood higher in his community. No man has done more for his fellow man. Countless numbers can rise up and call him blessed. And we may truly say, 'his compassion failed not even in the most trying occasions.' In the prolonged suffering endured by him courage and cheerful endurance characterized all the days and the end was peace. To God's gracious protection we commit him. The Lord bless him and keep him. The Lord lift up the light of His countenance upon him and give him joy in His eternal kingdom, through Jesus Christ, our Lord. Amen."

BIOGRAPHICAL.—James Wharton McLaughlin was born in Ohio, September 7, 1840. Came South just prior to the Civil War, enlisted as a private soldier in Company D, First Kentucky Infantry (C. S. A.). He served through the entire war with Johnson, Jackson, Morgan and Forrest. Settled in La Grange, Texas, studied medicine, graduated at Tulane, New Orleans, in 1867. Located at Columbus. Met and married in September, 1867, Tabitha Bird Moore, of Fayette county, and returning to La Grange practiced medicine until 1869. He then removed to Austin, just forty years ago. He is survived by his wife, three sons, Drs. Bird McLaughlin, of New York; Dr. Cyrus McLaughlin, of California, and Dr. Jas. W. McLaughlin, Jr., of Austin, and three daughters, Evelyn, Minnie (now Mrs. Porter) and Frances, a miss of sixteen, and a brother, Dr. Frank P. McLaughlin, of Austin, one of the best known surgeons of the State.

RESOLUTIONS BY TRAVIS COUNTY MEDICAL SOCIETY.

WHEREAS, Dr. J. W. McLaughlin, who departed this life November 13, 1909, was for many years an eminent practitioner of medicine in this State, a former President of the Texas State Medical Association, one of the founders of the Travis County Medical Society and long one of its most faithful members; and,

WHEREAS, He was ever an earnest student, seeking always the advancement and uplifting of scientific medicine, and making many important contributions to medical knowledge; therefore be it

*Resolved,* That the Travis County Medical Society feels deeply the loss of one of its most beloved members, whose memory we shall cherish as an honored colleague, a devoted practitioner, a true friend;

*Resolved,* That we extend to his family in their bereavement our sincere sympathy, and our appreciation of the great life of their loved one; and be it

*Resolved further,* That these resolutions be spread upon the minutes of this Society, that a copy be sent to the bereaved family, and to the press.

GEORGE M. DECHERD, M. D.

F. E. DANIEL, M. D.

H. L. HILGARTNER, M. D.

## Figures and Tables

**Figure f1:**